# Cotton Yield Estimation Based on Vegetation Indices and Texture Features Derived From RGB Image

**DOI:** 10.3389/fpls.2022.925986

**Published:** 2022-06-15

**Authors:** Yiru Ma, Lulu Ma, Qiang Zhang, Changping Huang, Xiang Yi, Xiangyu Chen, Tongyu Hou, Xin Lv, Ze Zhang

**Affiliations:** ^1^Xinjiang Production and Construction Crops Oasis Eco-Agriculture Key Laboratory, College of Agriculture, Shihezi University, Shihezi, China; ^2^Aerospace Information Research Institute, Chinese Academy of Sciences, Beijing, China

**Keywords:** yield, UAV, RGB image, vegetation indices, texture feature

## Abstract

Yield monitoring is an important parameter to evaluate cotton productivity during cotton harvest. Nondestructive and accurate yield monitoring is of great significance to cotton production. Unmanned aerial vehicle (UAV) remote sensing has fast and repetitive acquisition ability. The visible vegetation indices has the advantages of low cost, small amount of calculation and high resolution. The combination of the UAV and visible vegetation indices has been more and more applied to crop yield monitoring. However, there are some shortcomings in estimating cotton yield based on visible vegetation indices only as the similarity between cotton and mulch film makes it difficult to differentiate them and yields may be saturated based on vegetation index estimates near harvest. Texture feature is another important remote sensing information that can provide geometric information of ground objects and enlarge the spatial information identification based on original image brightness. In this study, RGB images of cotton canopy were acquired by UAV carrying RGB sensors before cotton harvest. The visible vegetation indices and texture features were extracted from RGB images for cotton yield monitoring. Feature parameters were selected in different methods after extracting the information. Linear and nonlinear methods were used to build cotton yield monitoring models based on visible vegetation indices, texture features and their combinations. The results show that (1) vegetation indices and texture features extracted from the ultra-high-resolution RGB images obtained by UAVs were significantly correlated with the cotton yield; (2) The best model was that combined with vegetation indices and texture characteristics RF_ELM model, verification set *R*^2^ was 0.9109, and RMSE was 0.91277 t.ha^−1^. rRMSE was 29.34%. In conclusion, the research results prove that UAV carrying RGB sensor has a certain potential in cotton yield monitoring, which can provide theoretical basis and technical support for field cotton production evaluation.

## Introduction

Cotton is an important cash crop, providing one of the world’s best high-quality fiber and natural crops, serving as one of the largest raw material supplies in the textile industry (Khan et al., 2020). In order to reduce production costs, reduce farmers’ labor burden, and improve cotton harvest efficiency, cotton harvest efficiency, use of machine for cotton cultivation has expanded over large areas (Wu and Chen, 2015). Spraying defoliating agent is the key technology of mechanized cotton harvesting (Sun et al., 2020), and the amount of defoliating agent and spraying time had significant effect on cotton yield (Xin et al., 2021). Analysis of the yield variation of the cotton harvest period is very important to determine the harvest time and evaluate the productivity of cotton (Tedesco-Oliveira et al., 2020). Therefore, it is of great significance to estimate cotton yield quickly and accurately before the cotton harvest.

The traditional yield survey method is based on the experience of farmers or professionals, which is time-consuming, laborious, and is mainly based on fixed point destructive sampling, which has a certain degree of uncertainty and cannot accurately evaluate the distribution of cotton yield in the region (Çopur et al., 2010). In recent years, the combination of artificial intelligence and remote sensing technology has been widely applied in agriculture (Xu et al., 2021). At present, relevant scholars have also proposed various methods for cotton yield prediction, such as the use of a yield detector mounted on the cotton picker (Pelletier et al., 2019), yield estimation based on crop growth models (Masasi et al., 2020), and yield monitoring realized based on multi-source satellite data (Meng et al., 2019). Compared to traditional methods, remote sensing methods are more economical and effective when it comes to cotton yield monitoring. However, existing remote sensing for crop yield monitoring has shortcomings such as a large amount of data, difficulty in data processing, or limitations in terms of resolution. With the development of remote sensing technology, unmanned aerial vehicle (UAV) low-altitude remote sensing platforms have become increasingly popular in the development of precision agriculture (Tsouros et al., 2019), At present, unmanned aerial vehicles can carry more sensors, such as hyperspectral, thermal image, RGB images, and LiDAR (Maddikunta et al., 2021). Compared with satellite remote sensing, UAV remote sensing platforms have strong flexibility, low cost, small atmospheric impact, and relatively high spatial and temporal resolution. Relevant researchers have studied the relationship between remote sensing information obtained by drones and crop yields. Tao et al. (2020) showed that the partial least squares regression (PLSR) allows the accurate estimation of crop yield from hyperspectral remote sensing data, and the combination of the vegetation indices and plant height allows the most accurate yield estimation. Maimaitijiang et al. (2020) used a UAV equipped with three sensors (RGB, multispectral, and thermal) to obtain remote sensing data, and a deep neural network framework was used to achieve multi-modal data fusion, to forecast soybean yield and effectively improve the accuracy of the prediction model.

At present, digital imagery provides the easiest and most common image information. The cost of RGB information acquisition is also very low, and it has been widely used in crop monitoring (Yamaguchi et al., 2021). Among the sensors typically carried by UAVs, RGB cameras have the advantages of small size, high resolution, and simple operation. RGB imagery can record the brightness digital number (DN) value of red, green, and blue wave segments, and color space conversion can be carried out according to this, and vegetation indices can be calculated. Compared with a spectral image or multi-source data fusion, RGB imagery is associated with a small amount of data and is easy to process. It is more beneficial to reduce the cost and complexity of monitoring, by obtaining RGB images with a UAV and fully mining the image information. Previous studies have extracted vegetation indices from RGB imagery to realize crop monitoring. For example, cotton yield monitoring research has been based on unmanned aerial vehicle multi-sensor realization (Feng et al., 2020), extracting the boll number from RGB imagery (Yeom et al., 2018); the cotton pixels were then separated using image processing technique and k-means with 5 class (Maja et al., 2016), or base UAV visible light remote sensing images extracted boll opening pixel percentage, and vegetation indices during the blooming period were used for estimating single boll weight (Xu et al., 2020). Most of the existing researches are based on image processing technology and need large amount of calculation and high hardware requirements. However, direct monitoring of cotton yield through the use of the visible vegetation indices combined with texture features has been studied less.

Vegetation indices, calculated based on visible and near infrared spectra, will appear to be saturated when the vegetation coverage is high during the growth stage of crops (Yue et al., 2019). Vegetation indices constructed based on RGB imagery also encounter the same problem, as they are calculated based on the brightness values of the R, G, and B bands only, less information, and small changes in vegetation indices. RGB images can be subjected to color space conversion and texture feature calculation. In the current research, mostly in the ground scale are by converting the color space achieved background segmentation and classification (Mao et al., 2020; Riehle et al., 2020). But there is a lack of research on monitoring cotton yield with different color space models at the ground scale. In addition, considering the centimeter-level high-resolution RGB images obtained by UAVs, the fusion of texture features and color features can lead to information complementarity and extracting more meaningful information from the imagery. Previous studies have shown that image texture features extracted based on gray level co-occurrence matrix (GLMC) are effective in nitrogen content estimation (Zheng et al., 2020) and the classification of diseases (Kurale and Vaidya, 2018), the results of which showed good performance.

There have been a lot of studies on cotton yield estimation using low-altitude UAV remote sensing, but few of them have utilized deep mining for RGB images. However, in terms of crop growth monitoring, (Yue et al., 2019) high-resolution RGB images and texture features obtained by UAV were used to monitor wheat biomass. Fernandez-Gallego (Fernandez-Gallego et al., 2019) estimated wheat yield using visible vegetation indices and color space. Therefore, in this study, UAV was used to obtain high-resolution RGB images from which vegetation index, texture features were extracted and converted color space model, and their combination were used to estimate cotton yield before harvest. Provide technical support for mechanical cotton harvesting and accurate management.

## Materials and Methods

### Experimental Design and Yield Investigation

**Figure 1:**
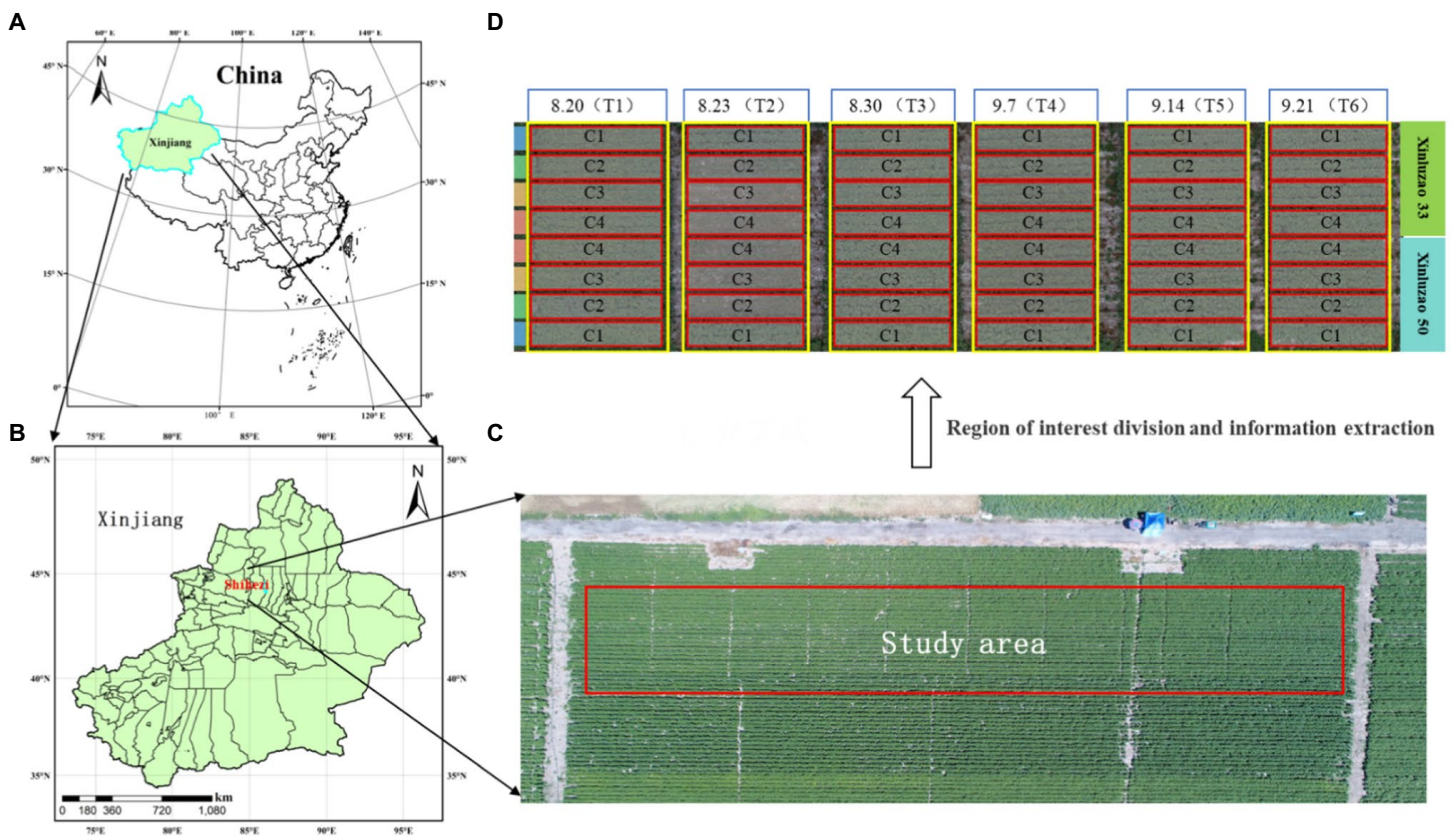
Study area and experimental design: cotton experiment at Shihezi University Teaching and Testing Ground, Shihezi, Xinjiang, China in 2019–2020. Experimental including two local varieties of cotton. C1, C2, C3, and C4 denote different defoliant concentrations. T1–T3 denote different sprayed times. **(A)** Geographical location of Xinjiang; **(B)** Geographical location of Shihezi; **(C)** Study area; **(D)** Experimental design.

For this study, a field experiment was carried out at the Shihezi University Teaching and Testing Ground, Shihezi, Xinjiang, China (Figure 1; 44°19′N, 85°59′E; altitude, 443 m). The study area is characterized by a temperate continental climate, in an arid and semiarid region, with an average annual precipitation of 125.9–207.7 mm, a large temperature difference between day and night, and a high soil nutrient content in the test area. Two local varieties of cotton (Xinluzao 50 and Xinluzao 33) were planted for two years (in 2019 and 2020). Six groups were planted per year, as shown in Figure 1D. Two cotton varieties, four defoliant concentrations, and six application time treatments were used to increase the yield differences. Taking Xinluzao 50 and Xinluzao 33, the main cotton varieties in the Xinjiang region, as the research objects, the defoliant concentration treatment included C1: clear water (CK); C2: defoliant 150 ml∙hm^−2^ + special additives 750 ml∙hm^−2^ + ethephon 1.2 l∙hm^−2^; C3: defoliant 300 ml∙hm^−2^ + special additives 750 ml∙hm^−2^ + ethephon 1.2 l∙hm^−2^; and C4: defoliant 450 ml∙hm^−2^ + special additives 750 ml∙hm^−2^ + ethephon 1.2 l∙hm^−2^. A defoliant (Total active ingredient content, 540 g∙L^−1^; diuron content, 180 g∙L^−1^; thidiazuron content, 360 g∙L^−1^, as a suspension agent) and special auxiliaries by the materials company Bayer AG production were used in this test. Defoliant was sprayed on August 20 (T1), August 23 (T2), August 30 (T3), September 7 (T4), September 14 (T5), and September 21 (T6) as shown in Figure 1D. Cotton was harvested after different defoliant spraying times and concentrations. Defoliant concentration tests (C1, C2, C3, and C4) of different varieties of cotton were carried out in the six experimental groups under the different spraying time treatments (T1, T2, T3, T4, T5, and T6). Theoretical yield investigation was carried out on the 3rd, 6th, 9th, 12th, and 15th days after the application of defoliant in the cotton opening period and before harvest. Yield per unit area was calculated by counting the number of bolls at the opening and the weight of a single boll in each plot. The single boll weight used in this study is the average weight calculated by selecting ten consecutive cotton plants in each plot (2.5 m*10 m), investigating their yield and boll number as given in equation (1).


(1) $$singlebollweight=\frac{10\;plantsyield}{10\;plantsbollnumber}$$

(2) Theoreticalyield=siglebollweight×bollnumber

### UAV Canopy RGB Image Data Collection and Processing

Before the cotton harvest, the Phantom 4 Advanced Aerial Photography UAV (Shenzhen, DJI, China) was used to capture high resolution color images of the entire experimental area. The drone image acquisition was done between 12:00 PM and 1:00 PM. During image acquisition, the camera (sensor size 5,472 × 3,648 pixels) was set to be vertically downward and set to equal time intervals. The flight height was set as 10 m above ground, with forward and side overlap set to 80%, the camera shutter time was 1/240 s, and the ISO value was 100. A total of 387 images were obtained from the experimental area. These images were stitched into orthophotographs with the Pix4D Mapper software (Pix4D, Switzerland), and stored in a TIFF format. The orthomosaics retain the gray-scale information of red, green, and blue colors of the ground objects. Each color contained 8-bit information, with a numerical range from 0 to 255. According to the plots distribution, the stitched cotton field image was cut into 48 regions of interest.

### Feature Extraction and Analysis of RGB Images

#### Extraction of Vegetation Indices

Some color space models and vegetation indices based on RGB were selected from previous studies (Table 1). The region of interest (ROI) is divided based on each test cell, and DN values of the three colors (red, green, and blue) were included for orthomosaics. We used MATLAB 2019A to obtain the DN values of three color channels, calculate the average DN value of each color channel, and calculate the normalized value of the three colors (R, G, and B), to calculate the vegetation indices. The equations used to calculate normalized values and vegetation indices are shown in Table 1.

**Table 1:** Review of the color space and vegetation indices used in this study.

**Data type**	**Band and vegetation indices**	**Equations**	**References**
Color space	R	DN value of the red band	
	G	DN value of the green band	
	B	DN value of the blue band	
	R	$r=\frac{R}{\left(R+G+B\right)}$	
	G	$g=\frac{G}{\left(R+G+B\right)}$	
	B	$b=\frac{B}{\left(R+G+B\right)}$	
	Y	Y=0.299R+0.587G+0.114B	
	Cb	Cb=0.568(B−Y)+128	
	Cr	Cr=0.713(R−Y)+128	
	U	U=0.493(B−Y2))	
Vegetation indices	NGRDI	NGRDI=(g−r)/(g+r)	Hamuda et al., 2016
	MGRVI	$MGRVI=\frac{\left(g^{2}-r^{2}\right)}{\left(g^{2}+r^{2}\right)}$	Bendig et al., 2015
	RGBVI	$RGBVI=\frac{\left(g^{2}-br\right)}{\left(g^{2}+br\right)}$	Bendig et al., 2015
	NDI	$NDI=\frac{\left(r-g\right)}{\left(r+g+0.01\right)}$	Hamuda et al., 2016
	VARI	$VARI=\frac{\left(g-r\right)}{\left(g+r-b\right)}$	Gitelson et al., 2002
	WI	$WI=\frac{\left(g-b\right)}{\left(r-g\right)}$	Bendig et al., 2015
	CIVE	CIVE=0.441r−0.881g+0.385b+18.78745	Kataoka et al., 2003
	GLA	$GLA=\frac{\left(2G-B-R\right)}{\left(2G+B+R\right)}$	Chianucci et al., 2016
	ExG	ExG=2g−b−r	Hamuda et al., 2016
	ExR	ExR=1.4r−g	Hamuda et al., 2016
	ExGR	ExGR=3g−2.4r−b	Hamuda et al., 2016
	GLI	$GLI=\frac{\left(2g-b-r\right)}{\left(2g+b+r\right)}$	Louhaichi et al., 2001
	NGBDI	$RGBVI=\frac{\left(g-b\right)}{\left(g+b\right)}$	Hunt et al., 2005

The color features of each divided ROI were converted from RGB color space model to HSV, and LA * B *, and YIQ were calculated based on corresponding functions in MATLAB. YCrCb model conversion formula are shown in Table 1.

#### Spatial Features: Texture Features Based on Gray-Scale Co-occurrence Matrix (GLMC)

Ultra-high-resolution images (ground resolution of 0.3 cm/pixel) were obtained from a UAV flying at an altitude of 10 meters above ground level. Four texture features were calculated from four different angles (0°, 45°, 90°, and 135°), based on the gray-level co-occurrence matrix (GLMC), and the average value of the four texture features was obtained. The three bands of the RGB image were calculated as gray values, and then, the texture features were calculated. The texture features were calculated using the following equations:


(3) $$Asm=\underset{i}{\sum }\underset{j}{\sum }P{\left(i,j\right)}^{2}\;$$

(4) $$Ent=\underset{i}{\sum }\underset{j}{\sum }P\left(i,j\right)logP\left(i,j\right)\;$$

(5) $$Con=\underset{i}{\sum }\underset{j}{\sum }{\left(i,j\right)}^{2}P\left(i,j\right)\;$$

(6) $$Cor=\frac{\sum_{i}\sum_{j}\left(i,j\right)P\left(i,j\right)-\mu_{x}\mu_{y}}{\sigma_{x}\sigma_{y}}\;$$

### Construction and Evaluation of Cotton Yield Estimation Model

**Figure 2:**
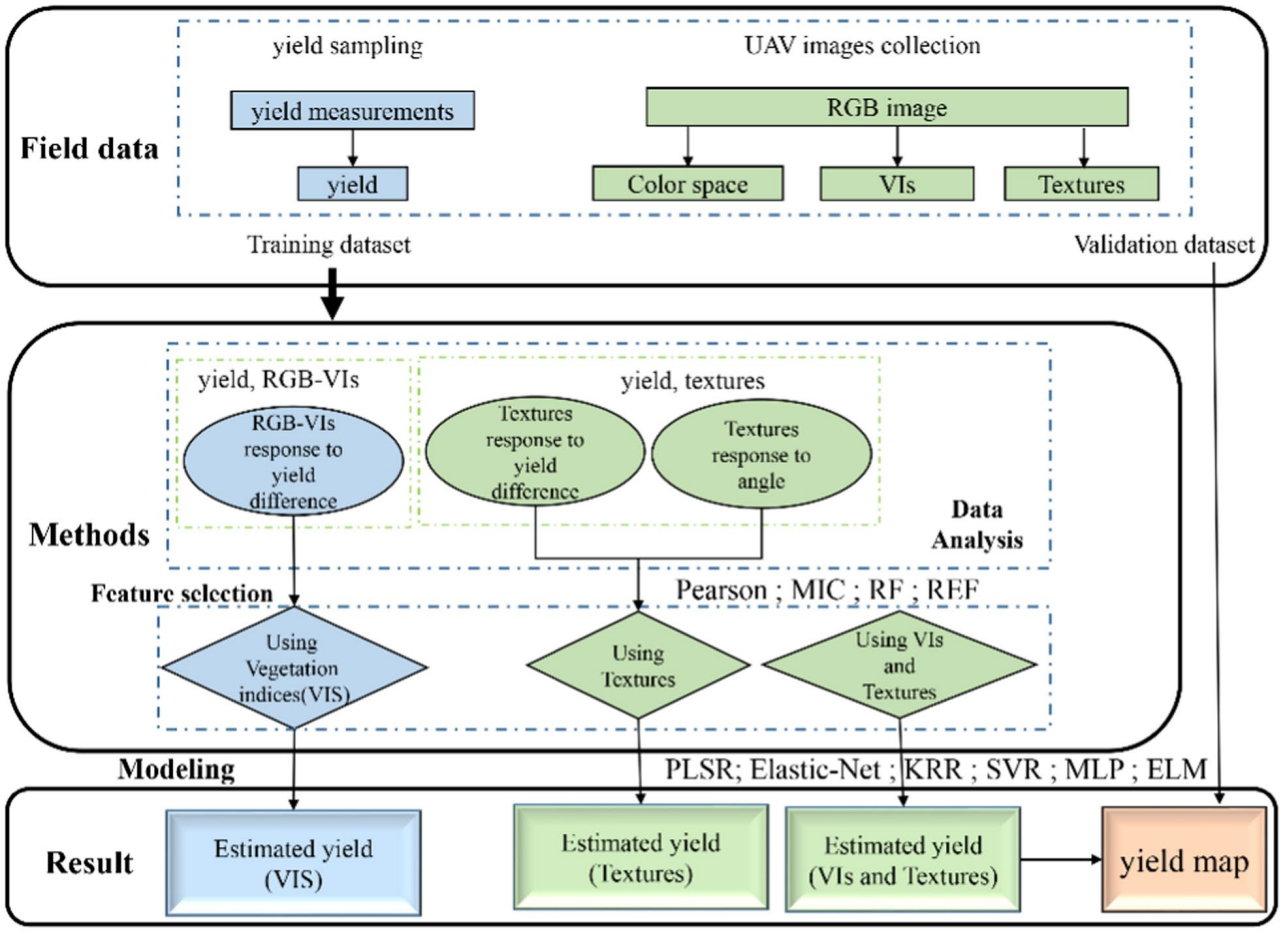
This study involves the flowchart of data acquisition, data processing, and model construction.

The flowchart shown in Figure 2 illustrates the experimental methods of this study, including field data collection, feature selection, model construction, and validation. In this study, vegetation indices and texture features, alone or combined, were used to estimate cotton yield. To further study the estimation accuracy of the yield estimation model, the cross-validation method was used to divide the data into training and validation datasets, with 315 samples for the 199 training dataset and 116 samples for the validation dataset. The descriptive statistics are shown in Table 2. There are significant differences between the data of training set and validation set.

**Table 2:** Cotton yield descriptive statistics.

**Dataset**	**Samples**	**Min (t.ha** ^**−1**^ **)**	**Mean (t.ha** ^**−1**^ **)**	**Max (t.ha** ^**−1**^ **)**	**Standard deviation (t.ha** ^**−1**^ **)**	**Coefficient of variation(%)**
Training set	199	0.00	3.00	11.58	2.91	97.08%
Validation dataset	116	0.00	3.08	9.83	2.75	89.25%

#### Optimal Feature Parameter Screening

The four methods of correlation, maximum information coefficient (MIC), random forest (RF), and recursive feature elimination (RFE) were adopted to select the optimal feature parameters for the model establishment, respectively, to eliminate the obvious collinearity among the parameters. The above methods are based on Python3.8 sklearn library. Parameter screening probability is to normalize the scores given by each method so that the values fall between 0 and 1.

#### Model

In the current research, the methods for establishing the relationship between remote sensing information and agricultural parameters mainly include three types: physical, statistical, and semiempirical models. In this study, a statistical model was established to achieve cotton yield monitoring. The statistical model mainly analyzed the correlation between the obtained UAV data and the measured output on the ground, and establishes regression models, including linear and nonlinear models. The linear regression model is fast and simple. Each characteristic variable can obtain a fixed weight, but the model structure is fixed and immutable. The nonlinear model is flexible, has a strong monitoring ability, and can eliminate certain collinearity; however, there is no fixed weight for each characteristic variable, it requires a large amount of data, takes a long time to calculate, and the output is a model framework, rather than a fixed formula. For this study, three linear regression methods were selected: partial least squares regression (PLSR), elastic neural network (Elastic Net), and kernel ridge regression (KRR). Three nonlinear regression methods were used to establish the cotton yield monitoring model: support vector regression (SVR), multilayer perceptron (MLP), and extreme Learning Machine (ELM). The above methods are based on Python3.8 sklearn library, and GridSearchCV of sklearn library is used to optimize model parameters.

#### Precision Evaluation

In this study, the coefficient of determination (*R*^2^), root-mean-square error (RMSE), and relative root-mean-square error (rRMSE) were used to evaluate the model performance. Higher *R*^2^ and smaller RMSE, rRMSE, denote higher model precision, accuracy, and stability. Those metrics were calculated as follows:


(7) $$R^{2}=\frac{\sum_{i=1}^{n}\left(x_{i}-\bar{x}\right)2\left(y_{i}-\bar{y}\right)2}{n\sum_{i=1}^{n}\left(x_{i}-\bar{x}\right)2n\sum_{i=1}^{n}\left(y_{i}-\bar{y}\right)2}\;$$

(8) $$\mathrm{RMSE}=\sqrt{\frac{1}{n}\overset{n}{\underset{i=1}{\sum }}\left(y_{i}-x_{i}\right)2}\;$$

(9) $$rRMSE=\frac{RMSE}{\bar{x}}\;$$

wherei represents the data of the ith sample point; $x_{i}$ is the measured value of the cotton yield for the ith sample point (in t.ha^−1^); $y_{i}\;$is the predicted cotton yield value at sample point i, estimated according to the model (in t.ha^−1^); $\bar{x}$ is the average value of measured cotton yield (in t.ha^−1^); and $\bar{y}$ is the average value of cotton yield estimated by the model (in t.ha^−1^).

## Results

### Relationship Between Vegetation Indices, Texture Characteristics, and Yield

**Figure 3:**
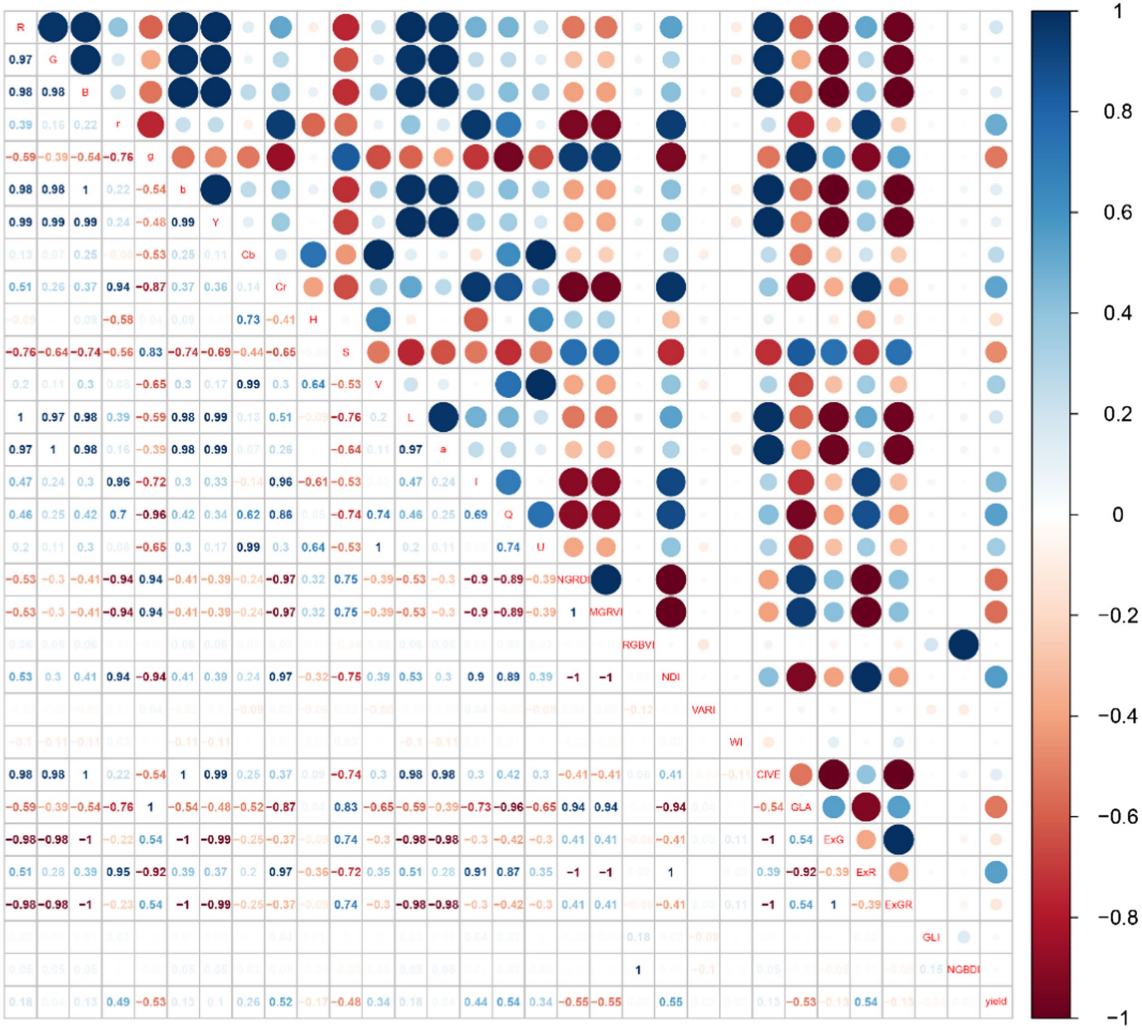
Correlation between different vegetation indices and cotton yield.

**Figure 4:**
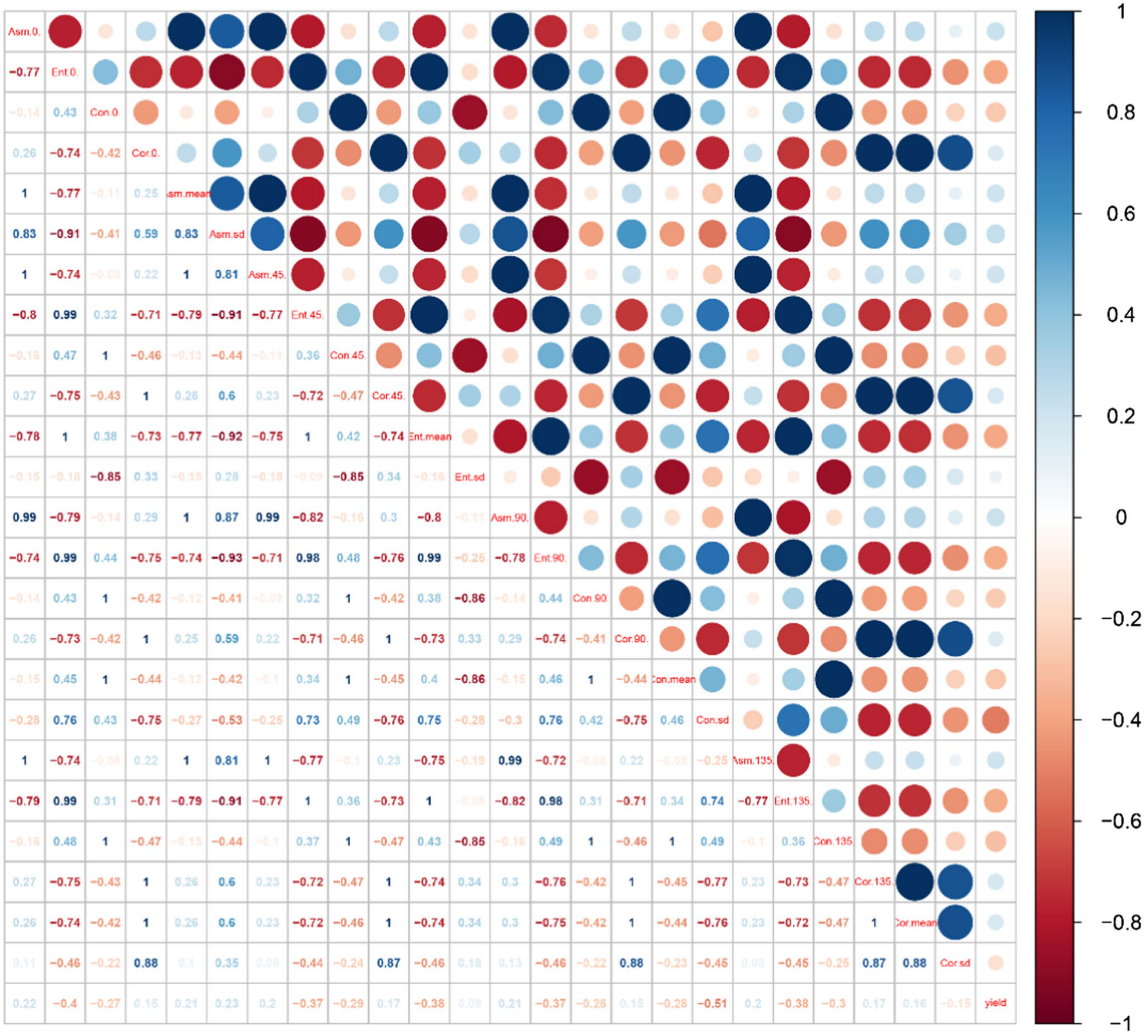
Correlation between different texture characteristics and cotton yield.

The red area represents a positive correlation, the blue area represents a negative correlation, and the lighter the color, the weaker the correlation. As shown in Figure 3, cotton yield was most correlated with NDI, NGRDI and MGRVI, with the correlation being 0.55, −0.55, and − 0.55, respectively. But there is serious collinearity among the three. As shown in Figure 4, cotton yield is positively correlated with ASM and COR at different angles, and negatively correlated with Ent and Con at different angles. The correlation with CON-SD was −0.51, indicating that with the increase in yield, the difference of Con in different directions became smaller and smaller. The distribution of image texture features is more and more uniform. Collinearity between texture features is also strong.

### Feature Selection

**Figure 5:**
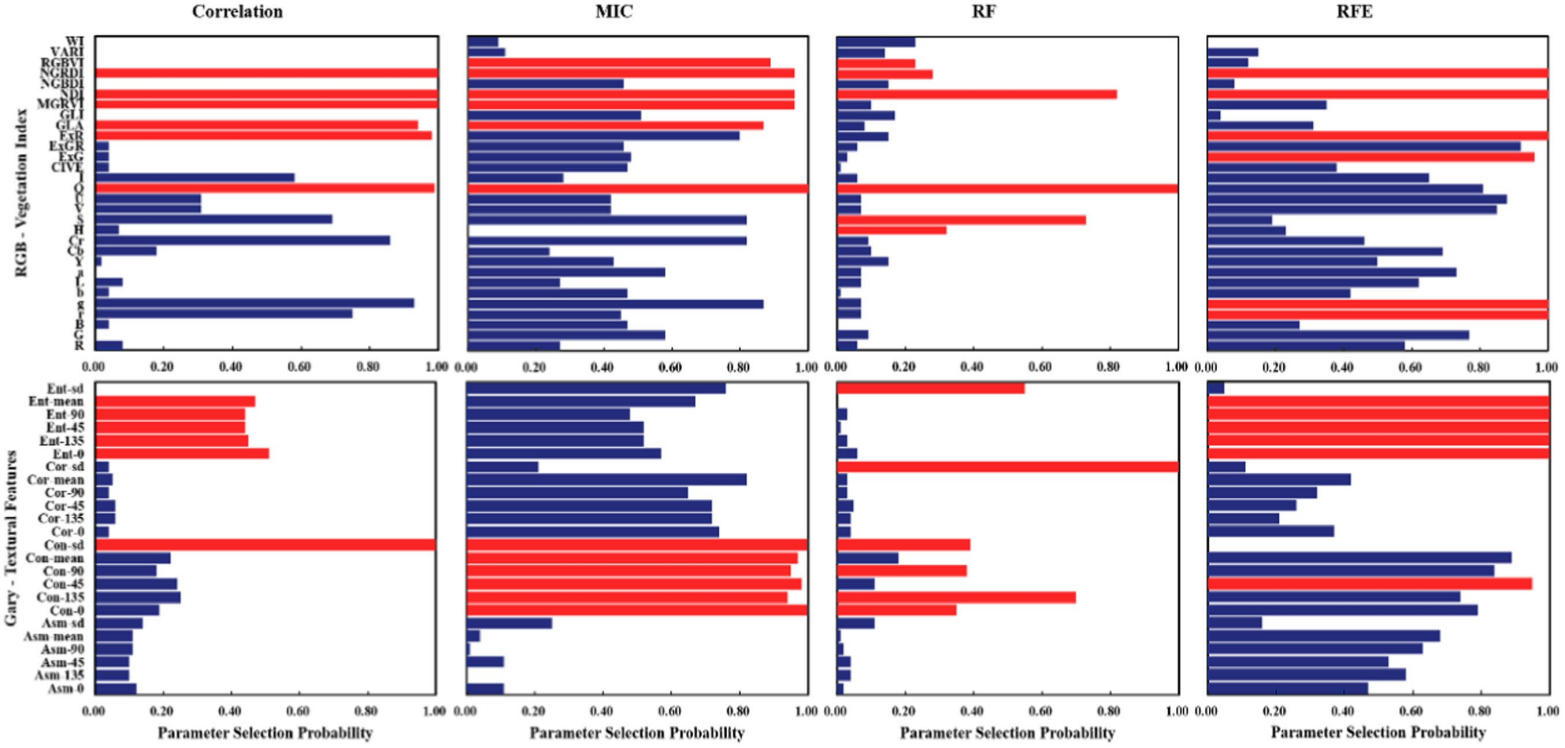
Optimal feature selection based on different screening methods.

To effectively eliminate the collinearity among variables and accurately estimate cotton yield, the optimal features were found from vegetation indices and texture features, based on the correlation coefficient, MIC, RF, and RFE. Then, different machine learning models were established, based on the six optimal features. Figure 5 shows the screening results of each method, in which the red bar indicates the optimal feature selected, and the feature parameters selected from each screening method are different. In combination with Figures 3, 4, it can be seen that the correlation between the characteristic parameters selected by RF was the lowest.

### Estimation of Cotton Yield Based on Vegetation Indices

**Figure 6:**
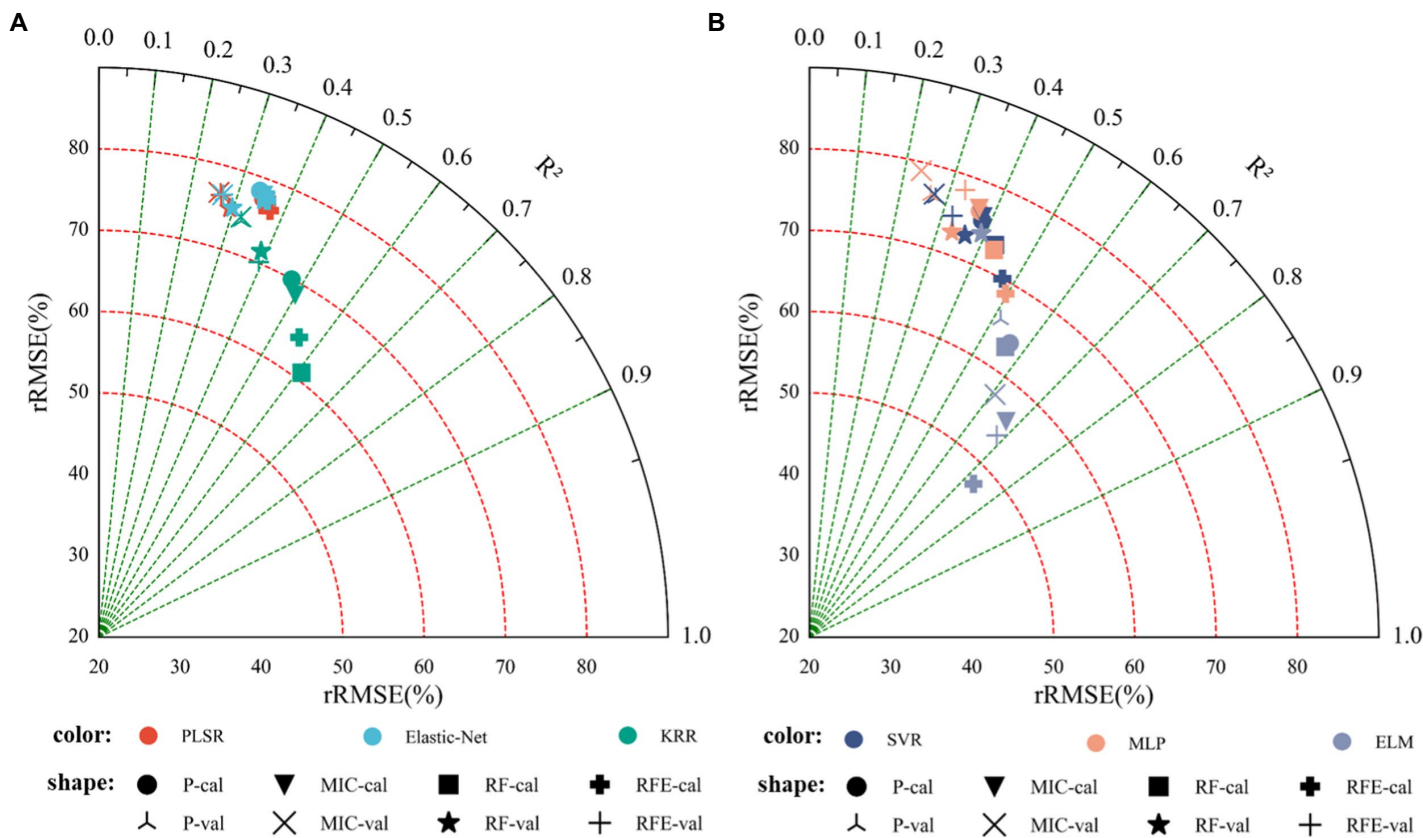
Monitoring model result of cotton yield based on the visible vegetation indices (Note: P denotes feature parameters screened out based on Pearson’s correlation coefficient, cal denotes training set; val denotes validation set. **(A)** Modeling results based on linear regression, **(B)** modeling results based on nonlinear regression).

**Figure 7:**
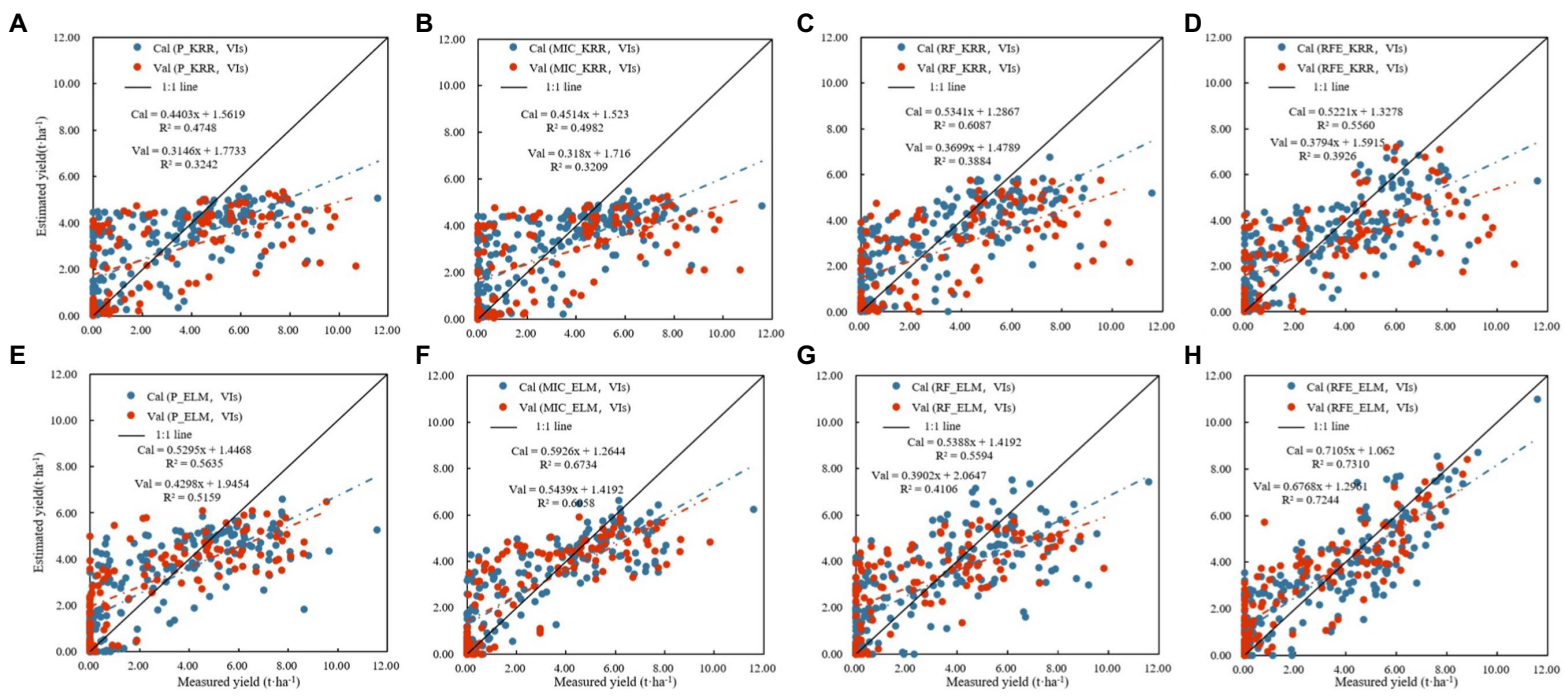
Cotton yield estimation models established by best performing based on the visible vegetation indices by using the linear and nonlinear models (Note: **(A)** P_KRR model; **(B)** MIC_KRR model; **(C)** RF_KRR model; **(D)** RFE_KRR model; **(E)** P_ELM model; **(F)** MIC_ELM model; **(G)** MIC_ELM model; **(H)** RFE_ELM model).

Table 3 shows the results of linear regression (PLSR, Elastic-Net, and KRR) and nonlinear regression (SVR, MLP, and ELM) monitoring models based only on vegetation indices. Figure 6 shows the fitting relationship between measured and predicted values of the model training and validation sets. The results showed that: (1) In the linear regression model, the RF_KRR model performed the best, the training set *R*^2^ was 0.6087, the RMSE was 1.7156 t.ha^−1^, and the rRMSE was 60.95%. But the verification set *R*^2^ only 0.3884, the RMSE was 2.4805 t.ha^−1^, and the rRMSE was 71.46%, among which. In the nonlinear regression models, the RFE_ELM model had the best effect. Training set *R*^2^ = 0.7310, RMSE = 1.4285 t.ha^−1^, rRMSE = 47.57%. The verification set *R*^2^ = 0.7244, the RMSE = 1.4418 t.ha^−1^, and the rRMSE = 49.23%, where the nonlinear models were better than the linear models, and the best model was the RFE_ELM model (2) Figure 7 shows the yield estimation model based on vegetation indices underestimated the high yield samples; the higher the yield, the worse the estimation ability. RFE_ELM effectively improved the estimation accuracy of high yield samples and, thus, improved the accuracy of the model.

**Table 3:** Cotton yield estimation based on vegetation indices.

Feature selection	Data set	PLSR			Elastic-Net			KRR			SVR			MLP			ELM		
		R^2^	RMSE (t.ha^−1^)	rRMSE (%)	*R* ^2^	RMSE (t.ha^−1^)	rRMSE (%)	*R* ^2^	RMSE (t.ha^−1^)	rRMSE (%)	*R* ^2^	RMSE (t.ha^−1^)	rRMSE (%)	*R* ^2^	RMSE (t.ha^−1^)	rRMSE (%)	*R* ^2^	RMSE (t.ha^−1^)	rRMSE (%)
P	Cal	0.35	2.1833	77.56	0.34	2.2054	78.35	0.47	1.9694	69.96	0.38	2.1379	75.45	0.37	2.1512	76.42	0.56	1.8191	63.70
	Val	0.26	2.6585	76.59	0.27	2.6521	76.41	0.32	2.5701	74.04	0.28	2.6446	76.19	0.26	2.6484	76.30	0.52	2.0215	65.55
MIC	Cal	0.36	2.1768	77.33	0.35	2.1960	78.01	0.50	1.9288	68.52	0.38	2.1374	75.93	0.3	2.1600	76.73	0.67	1.5799	55.89
	Val	0.26	2.6597	76.62	0.27	2.6538	76.46	0.32	2.5866	74.52	0.27	2.6573	76.56	0.23	2.7400	78.94	0.61	1.7314	57.55
RF	Cal	0.36	2.1675	77.00	0.36	2.1836	77.57	**0.61**	**1.7156**	**60.95**	0.43	2.0644	73.34	0.43	2.0475	72.74	0.56	1.8812	63.04
	Val	0.29	2.6074	75.12	0.30	2.6120	75.25	**0.39**	**2.4805**	**71.46**	0.36	2.5325	72.96	0.33	2.5268	72.80	0.41	2.1842	69.46
RFE	Cal	0.37	2.1531	76.49	0.36	2.1819	77.51	0.56	1.8106	64.32	0.47	1.9710	70.02	0.50	1.9305	68.58	**0.73**	**1.4285**	**47.57**
	Val	0.27	2.6515	76.39	0.27	2.6559	76.52	0.39	2.4342	70.13	0.32	2.5926	74.69	0.33	2.5404	78.19	**0.72**	**1.4418**	**49.23**

The values in bold represent models with the best results in linear and nonlinear methods.

### Cotton Yield Estimation Based on Texture Features

**Figure 8:**
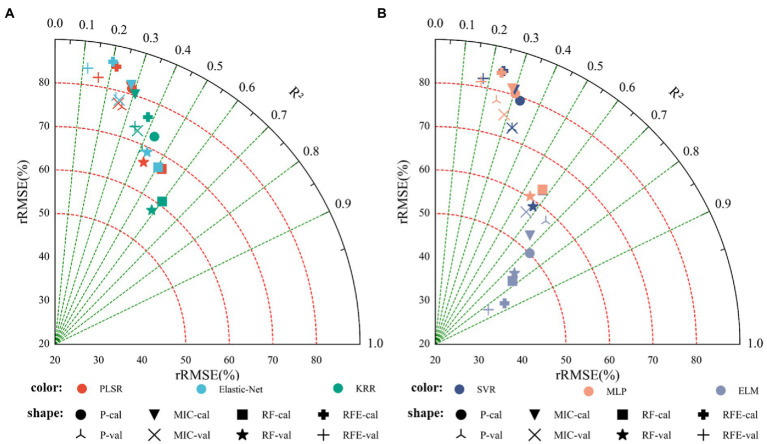
Cotton yield monitoring model based on texture features. **(A)** Modeling results based on linear regression, **(B)** modeling results based on nonlinear regression.

**Figure 9:**
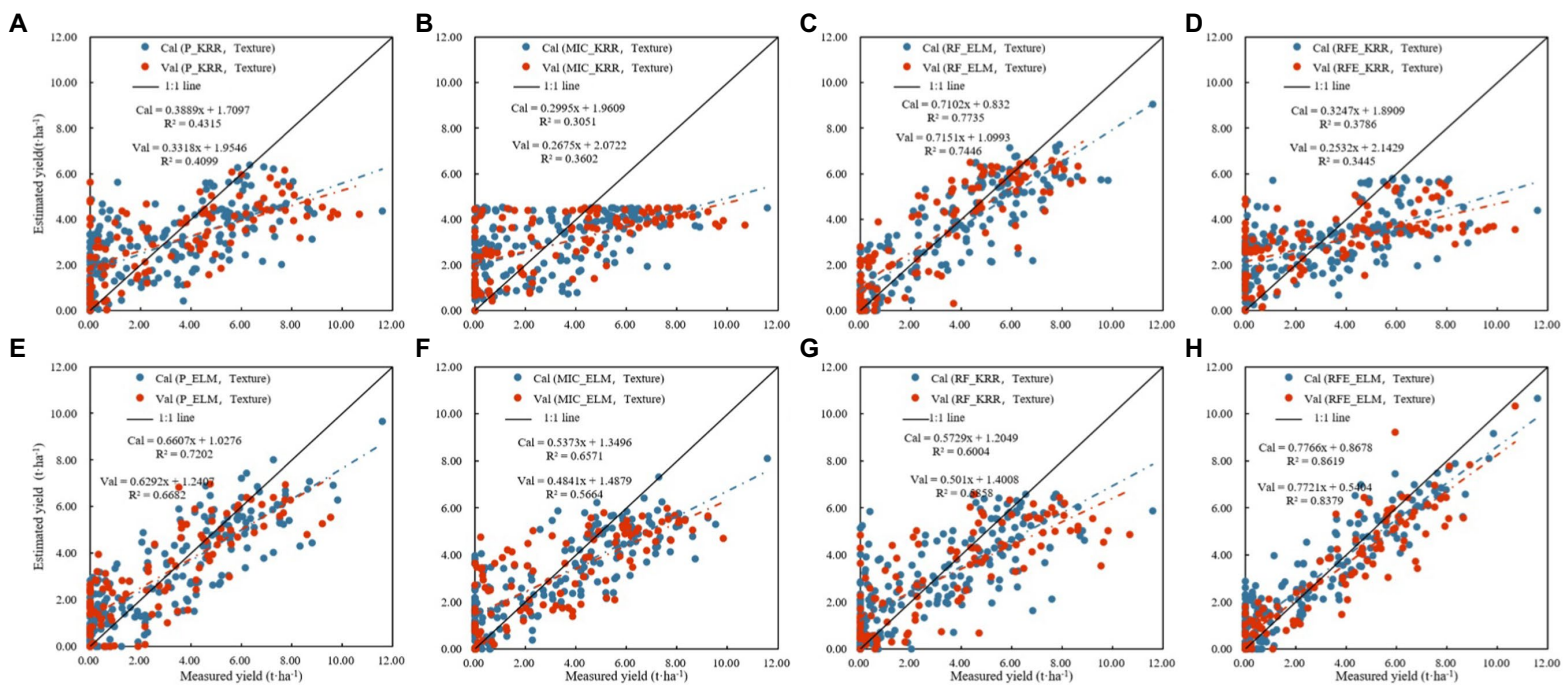
Cotton yield estimation models established by best performing based on the texture features by using the linear and nonlinear models. **(A)** Modeling results based on linear regression, **(B)** modeling results based on nonlinear regression.

Table 4 shows the *R*^2^, RMSE, and rRMSE of the monitoring model using only texture features, based on linear regression (PLSR, Elastic-Net, KRR) and nonlinear regression (SVR, MLP, ELM) methods. Figure 8 shows the results indicated that: (1) The optimal linear regression model was the RF_KRR model (training set *R*^2^ = 0.6004, RMSE = 1.7164 t.ha^−1^, rRMSE = 60.97%; verification set *R*^2^ = 0.5858, RMSE = 2.0112 t.ha^−1^, rRMSE = 57.97%), while the optimal nonlinear regression model was the RFE_ELM model (training set *R*^2^ = 0.8619, RMSE = 1.0941 t.ha^−1^, rRMSE = 38.46%; verification set *R*^2^ = 0.8379, RMSE = 1.1705 t.ha^−1^, rRMSE = 34.54%). Furthermore, the best nonlinear model was better than the best linear model (2) Figure 9 shows fitting relationship between the predicted and measured values of the training set and the verification set of the monitoring model. The yield estimation model based on texture features underestimated the high yield samples, but the effect was better than that of the yield estimation model based on vegetation indices.

**Table 4:** Cotton yield estimation results based on texture features.

Feature selection	Data set	PLSR			Elastic-Net			KRR			SVR			MLP			ELM		
		*R* ^2^	RMSE (t.ha^−1^)	rRMSE (%)	*R* ^2^	RMSE (t.ha^−1^)	rRMSE (%)	*R* ^2^	RMSE (t.ha^−1^)	rRMSE (%)	*R* ^2^	RMSE (t.ha^−1^)	rRMSE (%)	*R* ^2^	RMSE (t.ha^−1^)	rRMSE (%)	*R* ^2^	RMSE (t.ha^−1^)	rRMSE (%)
P	Cal	0.29	2.2891	81.32	0.28	2.3116	82.12	0.43	2.0515	72.88	0.33	2.2290	79.19	0.30	2.2644	80.44	0.72	1.4666	50.09
	Val	0.27	2.6575	76.56	0.24	2.7222	78.43	0.41	2.3907	68.88	0.33	2.5235	72.70	0.24	2.6975	77.72	0.67	1.5518	57.96
MIC	Cal	0.28	2.2989	81.67	0.28	2.3107	82.09	0.31	2.2599	80.28	0.30	2.2868	81.24	0.29	2.2897	81.34	0.66	1.7020	53.09
	Val	0.25	2.6807	77.23	0.25	2.7053	77.94	0.36	2.5190	72.57	0.33	2.5240	72.72	0.29	2.6043	75.03	0.57	1.8765	56.86
RF	Cal	0.52	1.8897	67.13	0.50	1.9702	67.00	**0.60**	**1.7164**	**60.97**	0.57	1.7760	63.09	0.57	1.7789	63.19	0.77	1.3601	42.93
	Val	0.44	2.3066	66.45	0.43	2.3906	68.87	**0.59**	**2.0112**	**57.94**	0.58	2.0398	58.77	0.54	2.0940	60.33	0.74	1.4065	44.42
RFE	Cal	0.22	2.4007	85.28	0.20	2.4267	86.21	0.38	2.1502	76.38	0.24	2.3826	84.68	0.24	2.3685	84.14	**0.86**	**1.0941**	**38.46**
	Val	0.16	2.8485	82.07	0.12	2.9104	83.85	0.34	2.5443	73.30	0.18	2.8473	82.03	0.17	2.8186	81.20	**0.83**	**1.1705**	**34.54**

The values in bold represent models with the best results in linear and nonlinear methods.

### Cotton Yield Estimation Based on Vegetation Indices and Texture Features

**Figure 10:**
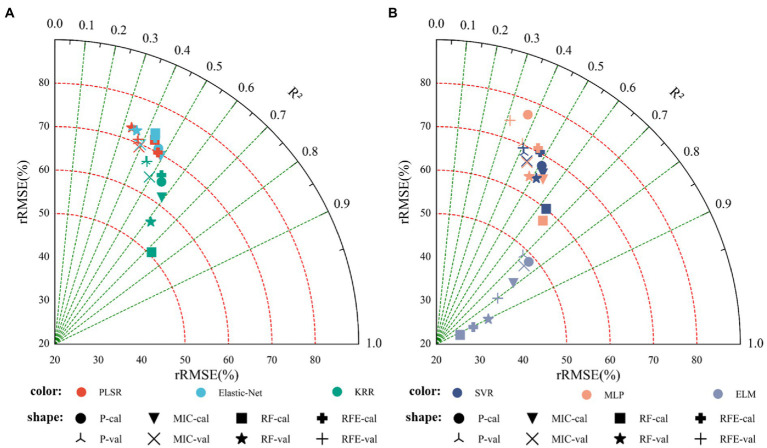
Cotton yield monitoring model based on the fusion of vegetation indices and texture features. **(A)** Modeling results based on linear regression, **(B)** modeling results based on nonlinear regression.

**Figure 11:**
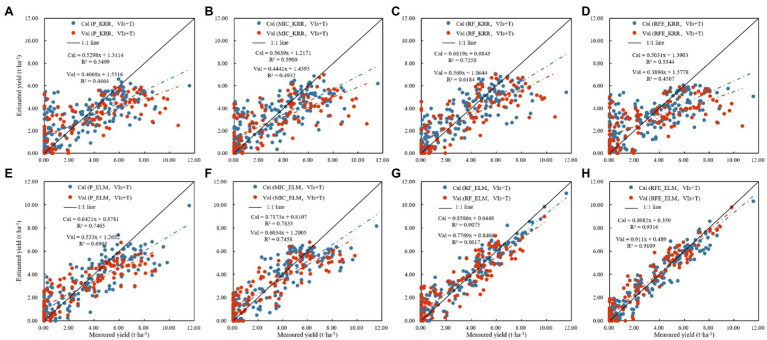
Cotton yield estimation models established by best performing based on the vegetation indices and texture features by using the linear and nonlinear models. (Note: **(A)** P_KRR model; **(B)** MIC_KRR model; **(C)** RF_KRR model; **(D)** RFE_KRR model; **(E)** P_ELM model; **(F)** MIC_ELM model; **(G)** MIC_ELM model; **(H)** RFE_ELM model).

Table 5 shows the results of establishing linear and nonlinear regression monitoring models by selecting the three vegetation indices and texture features with the highest selection probability as feature parameters. Figure 10 shows the results indicated that: (1) In the linear regression models, the training set *R*^2^ was 0.4306–0.7250, the RMSE was 2.0754–1.4283 t.ha^−1^, and the rRMSE was 73.73–50.74%, while the verification set *R*^2^ was 0.3348–0.6184, the RMSE was 2.5277–1.9348 t.ha^−1^, and the rRMSE was 72.82–55.74%, where the best model was RF_KRR. In the nonlinear regression models, the training set *R*^2^ was 0.3706–0.9316, the RMSE was 2.1631–0.7279 t.ha^−1^, and the rRMSE was 76.84–25.88%, while the verification set *R*^2^ was 0.3947–0.9109, the RMSE was 2.4377–0.9127 t.ha^−1^, and the rRMSE was 70.23–29.34%, among which the best model was the RFE_ELM model (2) Figure 11 shows the fitting results of measured and predicted values on the training and validation sets for the monitoring model, the yield estimation model based on vegetation indices and texture features underestimated the high yield samples, but the effect was better than that based on vegetation indices.

**Table 5:** Cotton yield estimation results based on vegetation indices and texture features.

Feature selection	Data set	PLSR			Elastic-Net			KRR			SVR			MLP			ELM		
		*R* ^2^	RMSE (t.ha^−1^)	rRMSE (%)	*R* ^2^	RMSE (t.ha^−1^)	rRMSE (%)	*R* ^2^	RMSE (t.ha^−1^)	rRMSE (%)	*R* ^2^	RMSE (t.ha^−1^)	rRMSE (%)	*R* ^2^	RMSE (t.ha^−1^)	rRMSE (%)	*R* ^2^	RMSE (t.ha^−1^)	rRMSE (%)
P	Cal	0.47	1.9824	70.42	0.47	1.9971	70.95	0.55	1.7202	64.66	0.51	1.9044	67.65	0.37	2.1631	76.84	0.75	1.4971	48.48
	Val	0.38	2.4432	70.39	0.38	2.4650	71.02	0.45	2.3235	66.94	0.42	2.3757	68.44	0.39	2.4377	70.23	0.70	1.6364	48.61
MIC	Cal	0.49	1.9446	69.66	0.49	1.9608	69.66	0.59	1.7382	61.75	0.53	1.8661	66.29	0.54	1.8334	65.13	0.78	1.3561	42.63
	Val	0.39	2.4048	69.28	0.40	2.4267	69.91	0.49	2.2275	64.17	0.44	2.3258	67.01	0.45	2.3142	66.67	0.75	1.5028	47.06
RF	Cal	0.44	2.0358	72.32	0.43	2.0754	73.73	**0.73**	**1.4283**	**50.74**	**0.63**	1.6923	60.12	0.65	1.6184	57.49	0.91	0.9277	29.34
	Val	0.33	2.5277	72.82	0.36	2.5190	72.57	**0.62**	**1.9348**	**55.74**	**0.52**	2.2403	64.54	0.49	2.2237	64.07	0.80	1.1699	37.64
RFE	Cal	0.47	1.9738	70.05	0.44	2.0503	72.76	0.53	1.8581	65.94	0.48	1.9775	70.18	0.46	1.9960	70.83	**0.93**	**0.7279**	**25.88**
	Val	0.38	2.4559	70.87	0.35	2.5136	72.54	0.45	2.3231	67.04	0.41	2.4028	69.34	0.31	2.5709	74.19	**0.91**	**0.8516**	**31.64**

The values in bold represent models with the best results in linear and nonlinear methods.

### Cotton Yield Inversion Map Based on UAV RGB Image

Based on the UAV RGB images covering 48 plots, vegetation indices and texture features were extracted, and the linear and nonlinear models with the best results (as described above) were selected for preharvest yield inversion. The results showed that the best linear model, RF_KRR, was able to monitoring the yields of <3.62 t.ha^−1^ accurately when estimating plots with low yield. However, as the yield increased gradually, the monitoring accuracy became lower and lower, and the yield of high-yielding plots was significantly underestimated such that yields greater than 8.33 t.ha^−1^ were not estimated. As for the RFE_ELM model, the estimation performance was better than that of the RF_KRR model, and its estimation results were similar to the measured values; however, yields <3.63 t.ha^−1^ were overestimated (Supplementary Figure 1).

## Discussion

At present, crop yield estimation based on remote sensing means is mainly realized based on satellite remote sensing data or unmanned aerial vehicles carrying multiple sensors. However, these methods have some limitations, mainly regarding the following aspects: (1) the resolution of satellite imagery is too course for that kind of application; and (2) multiple sensors acquire more information and, therefore, capture a large amount of redundant data, might require both large volume of hard drive space for storage and large amount of computer power to process the data. Therefore, for this study, we used an unmanned aerial vehicle carrying an RGB camera to obtain digital images with a high ground resolution and extracted vegetation indices and texture features to estimate cotton yield.

Crop yield monitoring is very important in crop management. Many studies have been devoted to using different methods to improve the accuracy of yield estimation models. Commonly used methods include establishing the model through vegetation indices (Zhang et al., 2020), multi-sensor monitoring (Feng et al., 2020), or deep learning (Escalante et al., 2019). RGB images are the most common type of image in daily life, which are characterized by simple acquisition and easy processing. More and more RGB images have been used for yield monitoring. Although these methods can effectively improve the accuracy of the yield estimation model, there is still room for improvement; for example, Liu et al. (2019) used the vegetation indices extracted from RGB imagery of a rice canopy to estimate its yield. The accuracy *R*^2^ of the model was improved, but only reached 0.7074. Fathipoor et al. (2019) used UAV RGB imagery to estimate forage yield, and the verification accuracy of their estimation model was 0.74, with a relative RMSE of 12.39%. In this paper, the RFE_ELM model was found to be the best model to estimate the yield through the use of vegetation indices, with *R*^2^ = 0.7310, RMSE = 1.4285, and rRMSE = 47.57% on the training set, and *R*^2^ = 0.7244, RMSE = 1.4418, and rRMSE = 49.23% on the verification set, similar to the results of (Huang et al., 2016), who used UAV RGB imagery to extract plant height and count the number of cotton bolls to estimate cotton yield. However, the accuracy was significantly lower than that of the cotton yield monitoring model established by Feng et al. (2020) using multi-sensor imagery. In this paper, the vegetation indices extracted from the UAV RGB imagery were significantly correlated with the cotton yield, such that it is feasible to select the optimal parameter features for cotton yield monitoring; however, this still needs to be improved from the perspective of model accuracy. Therefore, more texture feature information or other color information should be mined from RGB imagery, to improve model accuracy.

Texture features are an important parameter of RGB imagery, for which there exist many extraction methods. GLCM is the most commonly used and effective extraction method. In this paper, GLCM was used to obtain four texture features at different angles (0°, 45°, 90°, and 135°). Figure 2 shows that different cotton canopy grey values in the RGB imagery were used to extract texture features, some of which had significant correlations with cotton yield, where the difference had a significant correlation between different perspectives on the same parameters, namely according to different cotton canopy RGB image texture feature dependencies. This differed from the results of Zhang et al. (2017), in their study on satellite remote sensing texture feature extraction, and those of Zheng et al. (2020), who estimated nitrogen content in rice leaves by using an unmanned aerial vehicle to obtain multispectral images. As shown in Supplementary Figure 2, as we used a UAV with a flying height of 10 meters to capture high-resolution RGB images, no significant difference was found between each pixel, and we could select the ROI in an image, under the same processing. This was because where cotton planting density was higher, the canopy distribution was relatively uniform. However, in the study by Zhang et al. (2017), the satellite image separation rate was high, the difference between each pixel was obvious, and the distribution of image feature information was uneven. In the study of Zheng et al. (2020), the UAV flew at a height of 100 m and the selected area was multi-row, such that there was a difference in crop distribution due to the planting mode and, so, there was an obvious correlation in a certain direction.

Compared with the three estimation models based on vegetation indices, texture features, and their fusion, as used in this paper, texture features showed better performance than vegetation indices in cotton yield estimation, where the best model was the ELM model. The *R*^2^ increased by 17.91%, the RMSE decreased by 23.41%, and the rRMSE decreased by 19.15%, while for the verification dataset, the *R*^2^ increased by 15.67%, the RMSE decreased by 18.82%, and the rRMSE decreased by 29.84%. However, the performance of the monitoring model based on vegetation indices and texture features was better than that based on either vegetation indices or texture features alone, and the ELM model still performed the best. Compared with the model based on vegetation indices, the modeling results for the combined model, in terms of the *R*^2^, RMSE, and rRMSE, were increased by 27.44, 49.04, and 45.60%, respectively. For the verification dataset, the *R*^2^ increased by 25.75%, the RMSE decreased by 40.93%, and the rRMSE decreased by 35.73%. In conclusion, using both vegetation indices and texture feature information for cotton yield estimation can improve the accuracy of the model. This was similar to the results of Guo et al. (2021a), who monitored wheat yellow rust by using vegetation indices and texture features, and Yue et al. (2019), who estimated winter wheat biomass by using texture features and vegetation indices derived from the gray-scale correlation matrix of canopy imagery. However, in these two studies, the estimation performance when using the vegetation indices was better than that with the texture features. The analysis of vegetation indices and texture characteristics can be sensitive to the growth period, as the cotton leaves fall off gradually, and a more prominent G component (green) change is observed in the image but, in the later growth stage, when the dry leaves fall off, the G component decreases to a constant, while the yield is still changing. Therefore, vegetation indices are insensitive to yield changes associated with such gradual changes. Therefore, models based on vegetation indices tend to underestimate high yield plots, while texture features are calculated based on image gray values, which can effectively reflect the changes in the image feature; however, the dependence on the G component is weak, and the sensitivity to the changes of yield in the later growth period is reduced (but is still better than when using vegetation indices). In this study, the vegetation indices and texture features were combined to compensate for the saturation of vegetation index when the yield was high by virtue of the accuracy of vegetation index texture features in color, so as to improve the accuracy of the model to a certain extent. In previous studies, Zhang et al. (2021) used texture features, color and vegetation index to estimate wheat growth parameters, and also used texture features to compensate for index saturation and effectively improve the accuracy of the model. Zhou et al. (2021) used drones to diagnose water stress in winter wheat. Zhang et al. (2022) also introduced the combination of texture features and vegetation index to improve the model accuracy in the study of maize leaf area index estimation by UAV. Combined with the results of this study, it is feasible to improve the accuracy of cotton yield monitoring model by combining vegetation index and texture feature. Combined with the results of this study, it is feasible to improve the accuracy of cotton yield monitoring model by combining vegetation index and texture feature. As shown in Supplementary Figure 3, among the models established by integrating different pre-treatment methods, the best linear model was the model established by the KRR method, while the best nonlinear model was the ELM model; among these two, the ELM model had the best performance. This was similar to the results of Guo et al. (2021b), who used machine learning methods to predict rice yield by integrating phenological and meteorological data.

In this study, cotton yield monitoring based on simple RGB image provides technical support for cotton production and harvest. However, the model constructed in this study still lacks universality in different regions, different years, and different data acquisition conditions. In the future, more data set optimization models will be added, while image processing technology will eliminate the environmental impact of data acquisition to improve the model accuracy.

## Conclusion

In this study, ultra-high-resolution UAV RGB images were used to monitor the cotton yield before harvest. Vegetation indices, color spaces, texture features, and their combination were used to estimate the cotton yield before harvest from the RGB imagery. The results indicated the following:

1. The vegetation indices and texture features extracted from the ultra-high-resolution RGB images obtained by UAVs were significantly correlated with the cotton yield and, as such, can feasibly be used in cotton yield monitoring.
2. Comparing the modeling methods of linear and nonlinear regression, the cotton yield estimation model established by the nonlinear regression method had higher accuracy and stronger stability.
3. Comparing the cotton yield monitoring models based on vegetation indices or texture features, their fusion can further improve the monitoring ability of the cotton yield estimation model. The best model was the RFE_ELM model, the verification dataset *R*^2^ = 0.9109, RMSE = 0.91277 t.ha^−1^, and rRMSE = 29.34%.

## Data Availability Statement

The raw data supporting the conclusions of this article will be made available by the authors, without undue reservation.

## Author Contributions

YM, XL, and ZZ involved in research idea and scheme. YM, LM, and TH involved in experiment design. YM, XY, and TH UAV involved in data acquisition and processing. YM, QZ, and LM involved in ground data acquisition and analysis. YM, LM, and CH involved in data processing, model building, and validation. CH, TH, XL, and ZZ involved in resources. YM involved in writing original draft preparation. YM, LM, ZZ, XL, and CH involved in writing—review and editing. CH, XL, and ZZ involved in funding acquisition. All authors have read and agreed to the published version of the manuscript.

## Funding

This study was supported by the National Natural Science Foundation of China (No. 42061658; 41971321), Key Research Program of Frontier Sciences, CAS (No. ZDBS-LY-DQC012), Key Scientific and Technological Research Program of XPCC (No.2020AB005), The Common Application Support Platform for Land Observation Satellites of China’s Civil Space Infrastructure (CASPLOS_CCSI).

## Conflict of Interest

The authors declare that the research was conducted in the absence of any commercial or financial relationships that could be construed as a potential conflict of interest.

## Publisher’s Note

All claims expressed in this article are solely those of the authors and do not necessarily represent those of their affiliated organizations, or those of the publisher, the editors and the reviewers. Any product that may be evaluated in this article, or claim that may be made by its manufacturer, is not guaranteed or endorsed by the publisher.

## Supplementary Material

Supplementary Figure 1 Cotton yield inversion based on 2020’s UAV RGB imagery: **(A)** make cotton yield inversion map based on measured yield; **(B)** make cotton yield inversion map based on the vegetation indices and texture features by KRR model; **(C)** make cotton yield inversion map based on the vegetation indices and texture features by ELM model.

Supplementary Figure 2 Difference of texture features under different treatments.

Supplementary Figure 3 Comparison of results of different modeling methods.
