# Supplementary figures and images for: Cotton Yield Estimation Based on Vegetation Indices and Texture Features Derived From RGB Image
Source: Front Plant Sci. 2022 Jun 15;13:925986. doi: 10.3389/fpls.2022.925986 (PMC9240637; doi:10.3389/fpls.2022.925986)

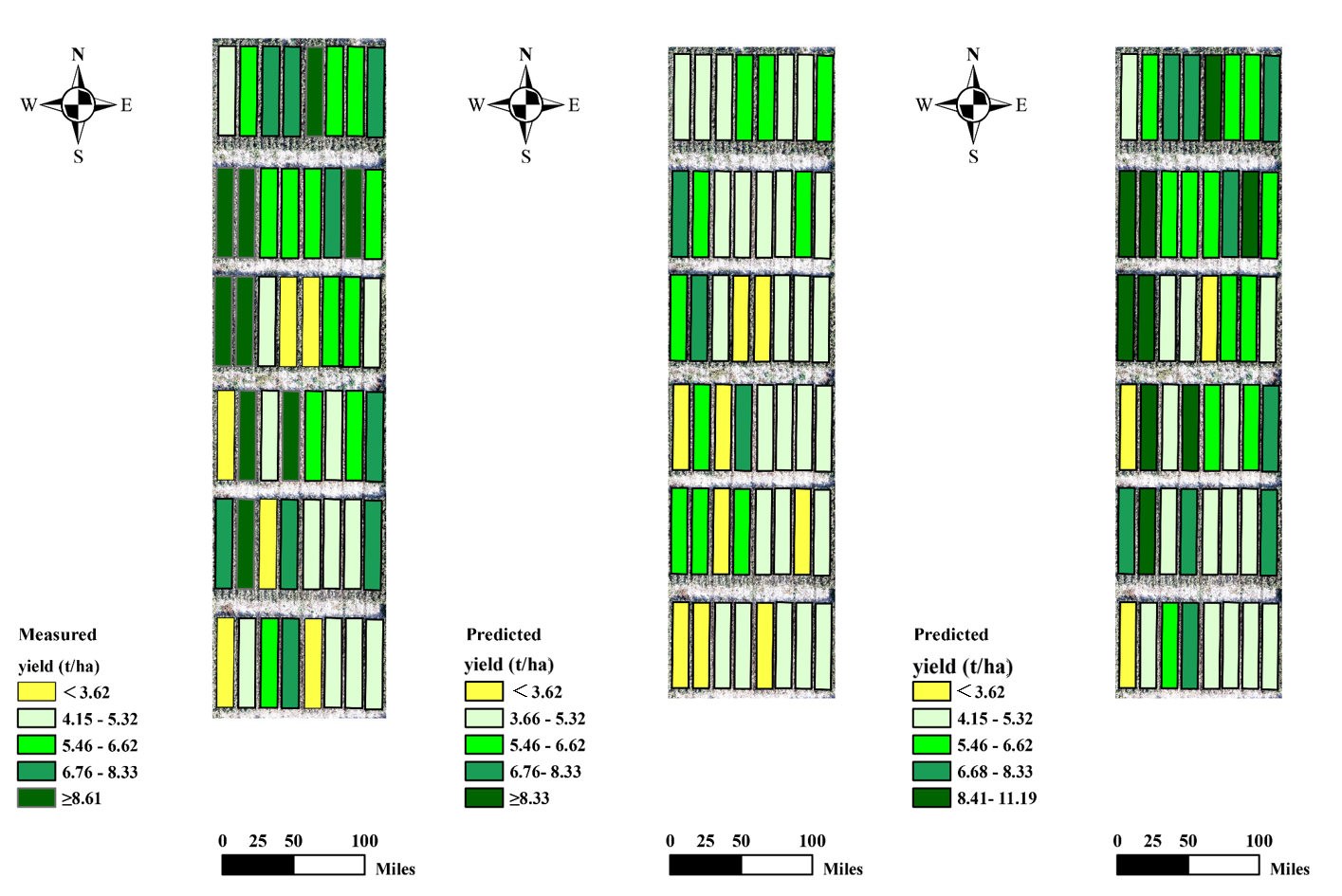

Supplement: Supplementary Figure 1 — Cotton yield inversion based on 2020’s UAV RGB imagery: (A) make cotton yield inversion map based on measured yield; (B) make cotton yield inversion map based on the vegetation indices and texture features by KRR model; (C) make cotton yield inversion map based on the vegetation indices and texture features by ELM model. [file Image_1.TIF]

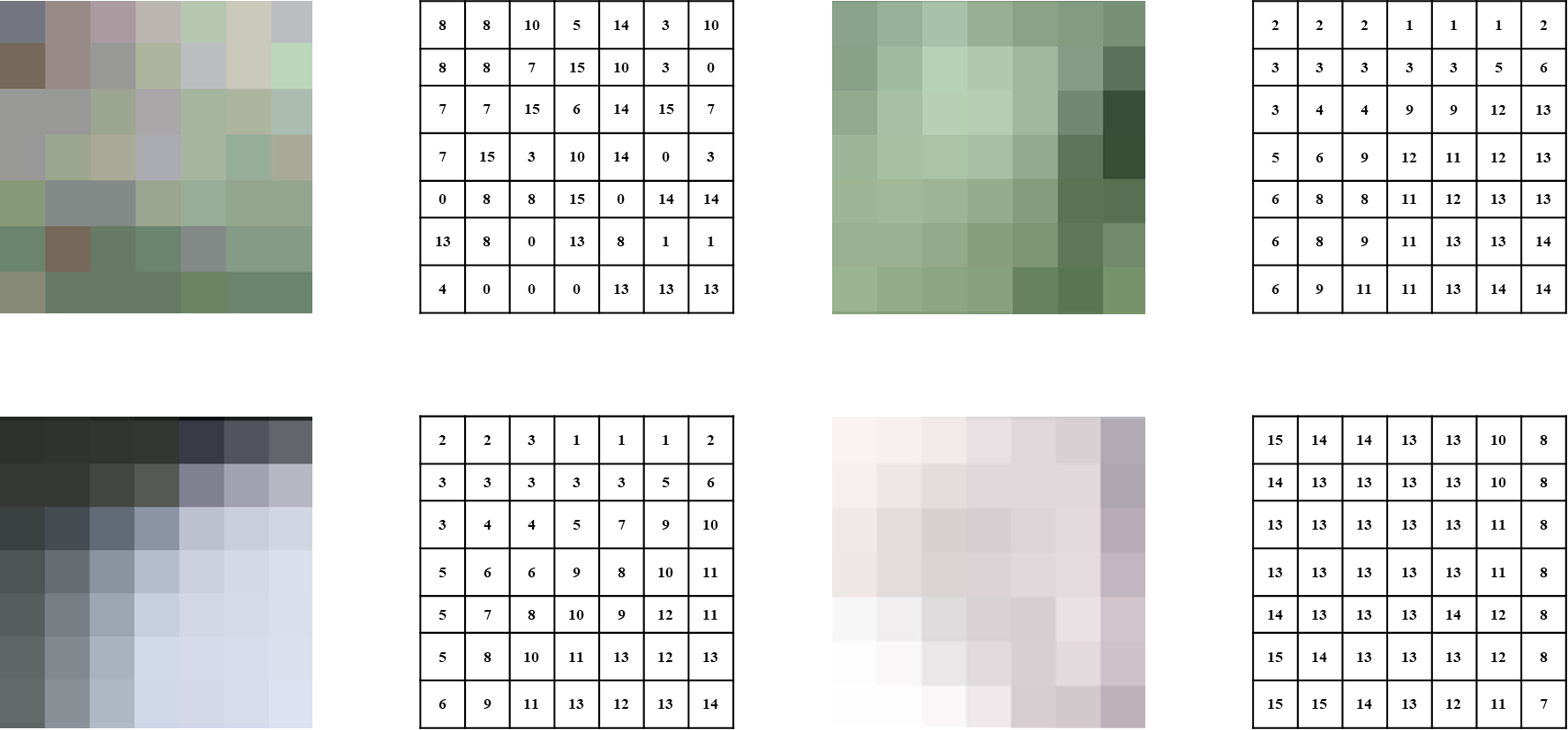

Supplement: Supplementary Figure 2 — Difference of texture features under different treatments. [file Image_2.TIF]

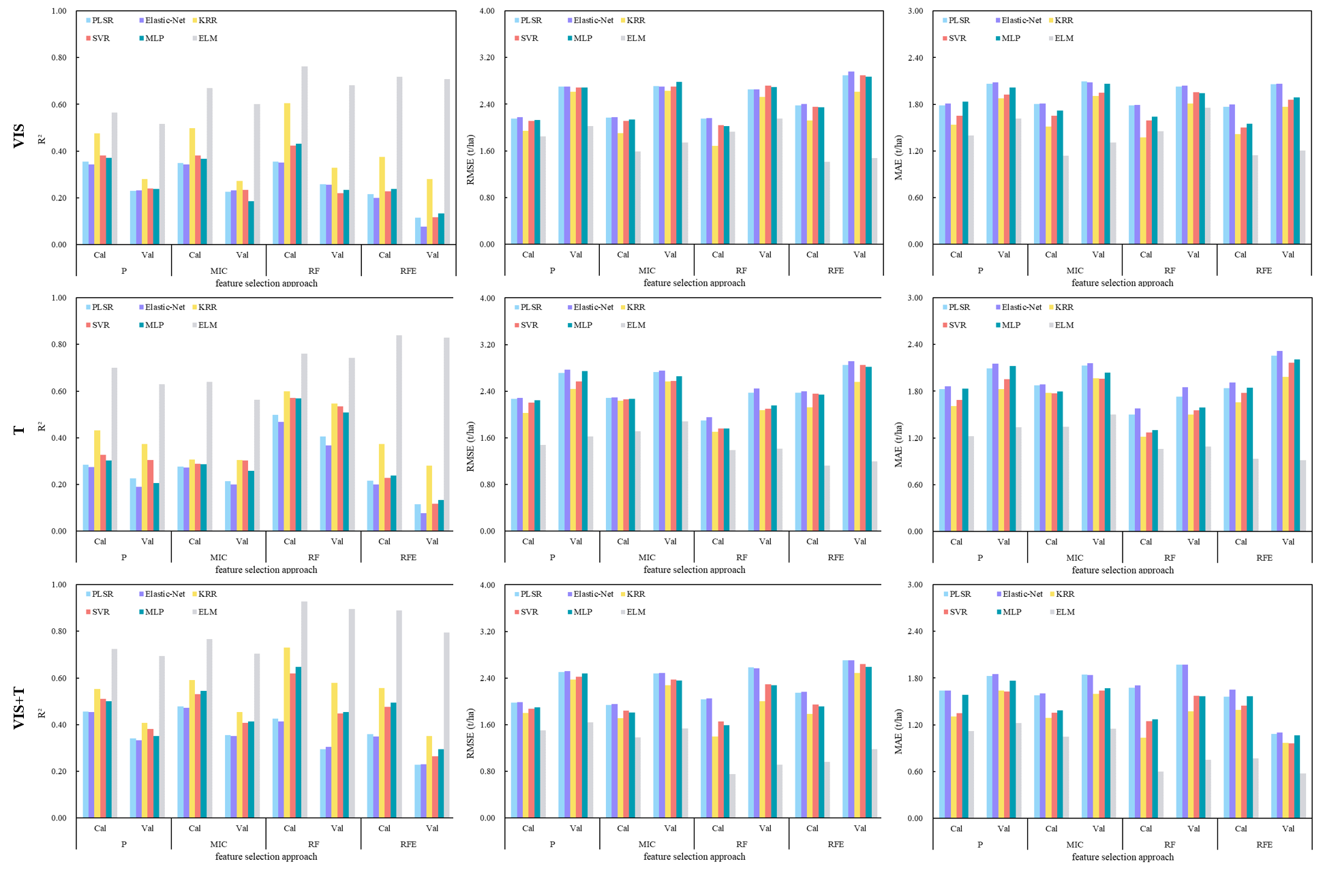

Supplement: Supplementary Figure 3 — Comparison of results of different modeling methods. [file Image_3.TIF]

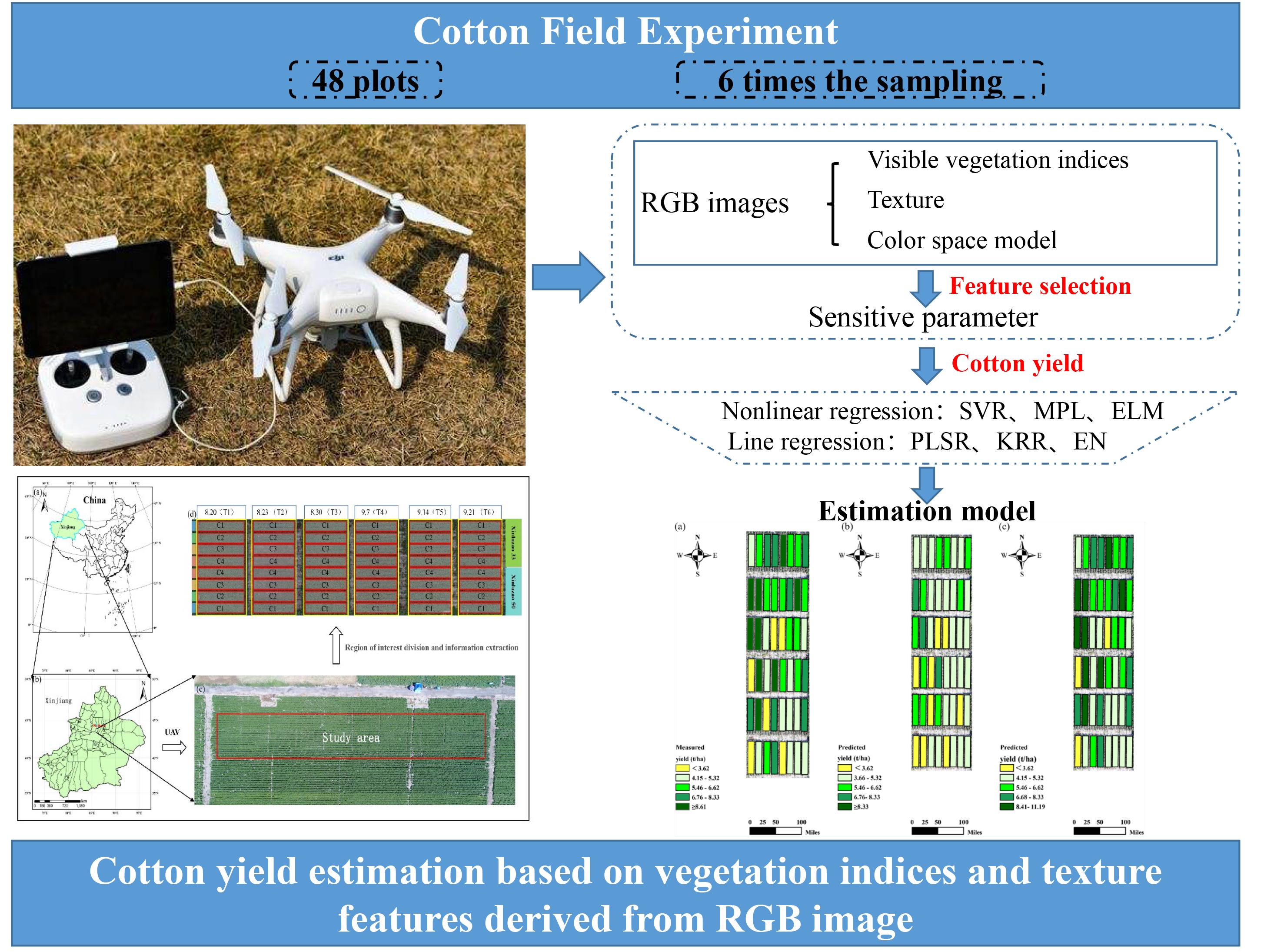

Supplement: Supplementary file 4 [file Image_4.TIF]
